# Influence of Chewing Ability on Elderly Adults’ Cognitive Functioning: The Mediating Effects of the Ability to Perform Daily Life Activities and Nutritional Status

**DOI:** 10.3390/ijerph19031236

**Published:** 2022-01-22

**Authors:** Yun-Sook Jung, Taejun Park, Eun-Kyong Kim, Seong-Hwa Jeong, Young-Eun Lee, Min-Jeong Cho, Keun-Bae Song, Youn-Hee Choi

**Affiliations:** 1 Department of Dental Hygiene, College of Science & Technology, Kyungpook National University, 2559, Gyeongsang-daero, Sangju-si 37224, Gyeongsangbuk-do, Korea; ysjung0313@gmail.com (Y.-S.J.); jinha01@naver.com (E.-K.K.); beijingjo72@hanmail.net (M.-J.C.); 2 Department of Preventive Dentistry, Kyungpook National University School of Dentistry, Daegu 41940, Korea; parktj@gmail.com (T.P.); kbsong@knu.ac.kr (K.-B.S.); 3 Faculty of Health Science, Daegu Haany University, 1, Hanuidae-ro, Gyeongsan-si 38610, Gyeongsangbuk-do, Korea; jeongsh@dhu.ac.kr; 4 Department of Dental Hygiene, Daegu Health College, 15, Yeongsong-ro, Buk-gu, Daegu 41453, Korea; yelee@dhc.ac.kr; 5 Institute for Translational Research in Dentistry, Kyungpook National University, Daegu 41940, Korea

**Keywords:** chewing ability, cognitive functioning, structural equation modeling

## Abstract

Chewing ability is also related to activities of daily living (ADLs) and nutritional status; however, these associations have not been firmly established. We examined chewing ability as a predictor variable and explored its relationship with cognitive functioning as mediated by ADLs and nutritional status data were collected by face-to-face interviews. Patients were receiving home healthcare service in Mun-gyeong city, Gyung-buk, Korea. Participants comprised 295 patients aged 81.35 ± 6.70 years. Structural equation modeling (SEM) was performed using AMOS 18.0 (SPSS Inc., Chicago, IL, USA). The model fit was based on absolute fit index and incremental fit index. Data were collected to assess cognitive functioning (using the Korean version of the Mini-Mental Status Examination for dementia screening (MMSE-DS)), ADL, a mini-nutritional assessment (MNA) questionnaire, and a chewing ability test. Participants with better chewing ability had significantly better cognitive functioning, ADLs, and nutritional status (*p* < 0.001). Chewing ability directly affected cognitive functioning and indirectly affected how ADLs and MNA affected MMSE-DS. Chewing ability is an important factor influencing the cognitive functioning of elderly adults in Korea, both directly and indirectly through mediating variables such as nutritional status and ADLs. Efforts to help older adults maintain their chewing ability are necessary for preventing cognitive impairment.

## 1. Introduction

The global demographic trend continues to age, with lower birth rates and increasing average life expectancy [1], and such trends are accelerating [2]. In 2015 among the entire population of Korea, 13.1% were elderly individuals aged > 65 years, representing 6,624,000 people and demonstrating an approximate increase of 2 million since 2006. It has been estimated that this figure will rise to 40% by 2060 [3]. The aging population of Asia, including Japan and Korea, is progressing rapidly. Due to the decrease in the typical “productive” working population (i.e., those aged 15–64 years), this could lead to economic burdens, an increase in elderly dependency costs, and increases in the demand for long-term care and welfare services due to chronic diseases such as stroke, dementia, and natural aging. This rapid increase in medical costs presents a serious constraint for both household and state coffers.

Dementia is a type of cognitive disorder most commonly observed in the elderly [4]. Such neuropsychological diseases occur because of damage to the nerve cells in the brain, leading to disabilities in memory, cognition, and overall social ability. Risk factors are multifactorial and include age, literacy, low educational levels, low socioeconomic status [5], injuries to the cephalic areas, obesity, smoking, high blood pressure, and diabetes, which are also risk factors [6]. Recent studies have also suggested that oral health factors, such as tooth loss, are risk factors [7].

Dental diseases also present an important problem for the elderly population, as oral health may decrease with aging. According to the 2008 National Health and Nutrition Examination Survey, only 53.6%, of elderly adults aged 65–75 years have 20 or more teeth (mean = 18 natural teeth), and 60% of them reported chewing disabilities [8]. Decreased chewing abilities lead to imbalances in nutritional status and decreased abilities to perform activities of daily living (ADLs) relating to physical health and bodily functions [7,9]. For the elderly, poor nutritional status is likely to lead not only to lower physical health but also to dementia. This is because the chronic diseases that are known to lead to dementia, such as deficiencies in vitamins, folic acid, and carotin; high cholesterol diseases; high blood pressure; and diabetes are related to nutritional disorders [10]. Moreover, ADLs relate to symptoms that occur prior to dementia, with ADLs decreasing significantly prior to the diagnosis of dementia [11]. In other words, decreases in chewing ability influence not only cognitive disorders but also nutrition and ADLs. Moreover, nutritional status and ADLs are related to cognitive disorders and are interrelated [12].

However, previous studies have focused on literature reviews [12], and most studies on conventional chewing ability have used bivariate analysis, analysis of variance, or multiple regression analysis. Structural equation modeling (SEM) is a strong multivariate analytical method that can express direct and indirect effects separately and show complex relations visually by using a path diagram. Therefore, it is suitable for analyzing what direct effect chewing ability has on cognitive functioning and whether it is indirectly affected by nutritional status and ADLs.

Cognitive disorders are even more serious, as they cannot be fully cured using currently available treatments, and they are also very difficult to prevent. Therefore, the appropriate method to manage dementia is to take proactive measures to prevent or slow the progression of dementia [13]. If oral health acts as the predictor of future dementia among elderly persons, managing the oral health status of this population, which is easier than managing dementia, may act as a method to prevent dementia. It is necessary to come up with the relationship between oral health and cognitive impairment through more diverse subjects and analysis methods. Consequently, the aim of this article is to identify both the direct effects of chewing ability on cognitive functions as well as the indirect effects mediated by ADLs and nutritional status.

The research hypotheses were as follows.

1. H1: Chewing ability will affect cognitive functioning;
2. H2: The ability to chew will affect the ability to perform everyday activities;
3. H3: Chewing ability will affect nutritional status;
4. H4: The ability to perform ADLs will affect cognitive functioning;
5. H5: Nutritional status will affect cognitive functioning;
6. H6: Nutritional status will affect the ability to perform daily activities;
7. H7: Chewing ability will indirectly affect cognitive functioning through the ADLs and nutritional status parameters;
8. H8: Mastication ability will indirectly influence the ability to perform daily activities through nutritional status parameters;
9. H9: Nutritional status will indirectly affect cognitive functioning through the parameters of ADLs;

## 2. Materials and Methods

### 2.1. Participants

This study was conducted with an elderly sample in Mun-gyeong city, Gyung-buk province, Korea. Elderly individuals aged > 60 years in Mun-gyeong city constitute 23% of the city population. As such, the city is very interested in job creation for elderly persons, retirement preparation and strategy, and ensuring welfare policies are actively developed. Moreover, the home help project for vulnerable and lower-income classes has been carried out by health centers in Mun-gyeong city This study was conducted among the elderly population who were receiving such home help services. A total of 310 participants were targeted; however, 295 (200 women) were included in the analysis, as six were excluded for not undergoing oral examinations or having missing survey sections.

### 2.2. Measurement Tools

The survey was conducted by seven visiting nurses who were trained before study initiation on the gum color judgment and the questionnaire method.

#### 2.2.1. Chewing Ability

##### Masticatory Performance Evaluating Gum

The gum we used to measure chewing ability was made by mixing glycerin-fat ester and micro-forming wax with an everyday gum base, making it like the gums in the market but not sticky like dentures. This Masticatory Performance Evaluating Gum (XYLITOL^®^; Lotte Co., Ltd., Saitama, Japan) includes xylitol, citric acid, and red, yellow, and blue coloring. The reliability and validity of this gum were verified by Kamiyama [14] in 2010, and it was found to be suitable for an epidemiological study in the elderly, as it simplifies measurement. Before chewing, the gum has a light green color; as the gum is chewed, the citric acid in the gum and saliva are released and mixed, which changes the color to red as the pH of the gum increases.

The measurement method was as follows. After the survey, participants who used dentures during meals were asked to chew the gum; those who did not use dentures were asked to chew the gum under the condition that was most like a meal condition. They were asked to chew on the Masticatory Performance Evaluating Gum for 2 min, and the color legend on the gum wrappings was used to measure performance after 2 min. Color changes were graded in five levels, with a darker pink color indicating a greater chewing ability. A scale ranging from 1 to 5 points was used, with light green representing 1 point and dark pink representing 5 points.

#### 2.2.2. Cognitive Abilities

##### Korean Version of the Mini-Mental Status Examination for Dementia Screening (MMSE-DS)

A leading tool for measuring cognitive functioning (dementia) in Korea and worldwide is the Mini-Mental Status Examination (MMSE) [15,16].

In 2009, the Ministry of Health and Welfare developed the new Korean version of the MMSE for Dementia Screening (MMSE-DS) along with corresponding guidelines [17,18]. The MMSE-DS is used to detect dementia in health centers across the country. Because the efficiency and reliability of the examinations were found to be adequate, we used the MMSE-DE as the dementia measuring tool in this study. Per the items suggested by Kim et al. [17], this tool constitutes 19 items, with subfactors including time orientation, spatial orientation, memory, focus and concentration, language abilities, execution abilities, time-spatial formation, judgments, and abstract reasoning. The scores ranged from 0 to 30, with lower scores demonstrating decreased cognitive functioning.

#### 2.2.3. ADLs

ADLs and instrumental ADLs (IADLs) were measured to determine whether participants required help from another person and to evaluate their functional status.

Katz developed the ADL scale in 1963 [19], which evaluates the most basic functions such as dressing, eating, ambulating, toileting, and bathing. This study used the Korean version of the ADL Scale (K-ADL), which was developed by Won [20] in 2002 and is suitable for conditions in Korea. The scores range from 7 to 21 points, with higher scores indicating higher dependency.

IADLs refer to activities required for independent living, consisting of decorating, housework, preparing meal, laundry, going out for a short distance, using transportation, shopping, handling money, using the telephone, and taking medicine. This study used the Korean version of the initial tool developed by Lawton and Brody in 1969 [21], which was modified by Won [20] to be suitable for the Korean culture (K-IADL). This scale is composed of 10 items, with scores ranging from 10 to 33 points; higher scores indicate greater dependency.

#### 2.2.4. Nutritional Status

We measured the nutritional status of the elderly subjects using the Mini-Nutritional Assessment (MNA), which was initially developed in 1994 by Vellas et al. [22] and was modified per the requirements of this study [23].

The MNA is divided into four sections with a total of 18 questions. The sections include subjective assessments of anthropometric measurements (body mass index, upper arm circumference, girth circumference, and weight loss), physical and psychological assessments (lifestyle, drug, mental stress or acute disease, behavioral ability, neuropsychiatric problems, bedsores, or ulcers), diet evaluations (frequency of meal intake, protein intake, fruit or vegetable intake, food intake, water intake, and self-feeding ability), and subjective assessments about health and nutrition. The MNA measures nutritional imbalances, with higher scores indicating fairer nutritional status.

### 2.3. Data Collection Methods

The study period was over 10 months from September 2014 to June 2015. Seven visiting care nurses visited the homes of participants to conduct the evaluations, and each nurse performed evaluations on approximately 40 elderly participants. Before the evaluations, the evaluators were trained and educated on the plan and the needs for this study. Training was provided on the survey methods of the MMSE-DS, MNA, and ADLs, as well as the ordering of oral health examinations. Nurses were trained for oral health examinations using the data provided by dentists and dental hygienists (Kappa > 0.7); guidelines for examinations were distributed for the nurses to adhere to when conducting examinations. 

### 2.4. Institutional Review Board (IRB)

The IRB approved the study protocol from the IRB (KNUH 2015-07-007-001) of the Kyungpook National University, and received consent from the elderly participants who voluntarily took part in this study. Before the start of the study, participants were told about the study, and if they agreed to participate, they signed the consent form. This study was conducted in accordance with the Declaration of Helsinki. 

### 2.5. Statistical Analysis

#### 2.5.1. Descriptive Statistics and Analysis of Variance (ANOVA)

We conducted descriptive statistical analysis for the data on demographic characteristics, chewing ability, ADLs, nutritional status, and cognitive functioning scores. We tested the statistical significance of the ADLs, nutritional status, and cognitive functioning scores depending on the chewing abilities of participants through one-way ANOVAs. When there were significant differences in the results, we performed Bonferroni post hoc evaluations to identify differences between groups in chewing function level.

Collected data were analyzed using IBM SPSS Statistics version 20.0 (IBA Corp., Armonk, NY, USA), and the significance level for statistical significance was set at 0.05.

#### 2.5.2. SEM Analysis

To measure the model fit of the study model and for hypothesis testing, we carried out a statistical analysis per the stages of SEM analysis. The collected data were subject to confirmatory factor analysis, goodness-of-fit tests, and hypotheses testing. First, the confirmatory factor analysis was carried out to ensure the validity and reliability of the measurement tools, and the convergent validity and discriminant validity were secured. Next, we evaluated the model’s goodness-of-fit by absolute indices of fit and incremental indices of fit; as the model fit met the standards of fit, the model was not subject to further modifications and was selected as the final study model. The testing of the hypothesis and of direct, indirect, and total effects were based on this final model. This study used the SEM statistical program AMOS 18.0 (SPSS Inc., Chicago, IL, USA).

## 3. Results

### 3.1. Characteristics of the Study Population

Table 1 presents the general characteristics of the participants. Among the study population, 67.8% were women, and the average age was 81.4 years.

### 3.2. ADLs, Nutritional Status, and Cognitive Functioning per Chewing Abilities and Their Respective Distribution

Among the surveyed items, the chewing function scores were distributed between 1 and 5 points. We classified these scores as follows: 1–2 points, low chewing function; 3 points, middle chewing function; and 4–5 points, high chewing function. We compared the ADL, MNA, and MMSE scores. Lower chewing functioning was related to higher dependencies in IADLs, lower nutritional assessment scores, and lower cognitive functioning (Table 2).

### 3.3. SEM Analysis

#### Validity Test

This study did not have issues of convergent validity, as the factor loading values (minimum = 0.5, >0.7) and the path coefficient (critical ratio [estimate/SE] values were larger than 1.965 and the *p*-values was smaller than 0.05), which all met respective standards. Next, regarding the discriminant validity of the latent variables used in this study, the ADL and cognitive functioning restricted model had ∆χ^2^ of more than 3.84, indicating significant differences in the model and that discriminant validity was present. This study assumes that the relationship between the nutritional status and ADL and cognitive functioning and ADL had negative relationships; in the correlations chart, they were indeed found to be negative, ensuring nomological validity.

### 3.4. Study Hypotheses Testing

**Figure 1:**
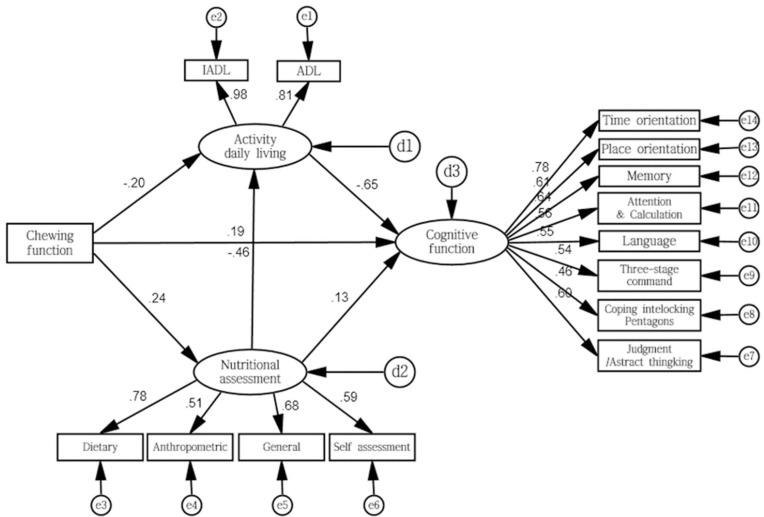
Path diagram illustrating the influence of chewing ability and cognitive function on elderly people: the mediating effects of the ability to perform daily life activities and nutritional status (*n* = 295). Rectangles represent observed variables, and ellipticals represent latent variables. Chewing function is the exogenous variable, and others are endogenous variables. Circles labeled e1–e14 indicate the measurement error of the corresponding observed variables, and d1–d3 indicate the structural error of the corresponding endogenous variable. Single-head arrows between exogenous variable and endogenous variable indicate hypothesized causal directions. Numbers attached to arrows between exogenous variable and endogenous are the standardized direct effect. The numbers attached to the arrows between latent variables and observed variables are standardized factor loadings on convergent validity (good convergent validity > 0.5).

Figure 1 shows the study model adjusted to the SEM model.

#### 3.4.1. Model Fit for the Study Model

The model fit is the difference between the covariance matrix of the collected sample data and the covariance matrix of the study model; this study used the model fit through the absolute index of fit and incremental index of fit. We conducted a structural analysis of covariance, with the model fit results shown in Table 3. In terms of the χ^2^ value, measuring the model fit using this value increases the sensitivity to the sample size and the number of measured variables, and the NC (normed χ^2^ = χ^2^/*df*) value is often used as its alternative. Normally, a value of <3 for the NC value is equated to the model being fit [24]. The NC value of this study was 2.037, with the model goodness-of-fit secured; therefore, we determined that the study model was appropriate for the sample data.

Chi-square statistic (*χ*^2^), normed *χ*^2^ (*χ*^2^/df). GFI, goodness-of-fit index; AGFI, adjusted GFI; CFI, comparative fit index; RMR, root mean-squared residual; RMSEA, root mean-squared error of approximation; TLI, Tucker–Lewis index; NFI, normed fit index.

#### 3.4.2. Path Coefficient for Study Model

The hypotheses were tested through the path coefficients between the variables forming the hypotheses (Table 4). All six hypotheses were confirmed, with statistical significance values of <0.05

#### 3.4.3. Direct, Indirect, and Total Effects

The hypothesis that chewing ability will indirectly influence cognitive functioning through the mediating variables of ADL and nutritional assessment had statistically significant direct, indirect, and total effects. H8, that chewing ability will indirectly influence ADLs through the nutritional assessment, had statistically significant direct, indirect, and total effects. Lastly, H9, that nutritional assessment will indirectly influence cognitive functioning through ADLs, did not have a statistically significant direct effect; however, the indirect and total effects were statistically significant (Table 5).

## 4. Discussion

Chewing ability is a core component of oral functioning. Nutrient intake is essential for sustaining human life, and it is even more important for the elderly, as it is closely related to prolonging life and maintaining health. Factors that decrease chewing abilities among elderly individuals were found to be tooth loss, decreased bite force, and saliva secretion rates [25,26], and the influences thereof are known to inflict nutritional disorders as well as disorders in ADLs and cognitive functioning [27,28].

Dementia begins with memory loss in its early stages (e.g., amnesia), followed by mental and action disorders in its middle stages and physical disorders in its late stages. Specific symptoms that follow cognitive functioning disorders and abnormal behavior render the understanding and adequate protection of elderly persons with dementia exceedingly difficult. Given this uniqueness, dementia leads to psychological and physical fatigue as well as economic crises for those supporting patients of dementia [29]. Moreover, dementia is a multifaceted disorder with strategies that can be used to combat it when detected in the early stages; however, most of these symptoms can be underestimated as simple forgetfulness and physical disabilities, owing to old age. As such, the prevention and early detection of dementia and other aging diseases are necessary; however, existing studies have focused on relationships between disorders from cognitive functioning and oral health [13,16,30,31], developing tools for measuring dementia [18,23,32,33], or the identification of actions, mental disorders, and risk factors of specific groups of elderly [13,29,34,35,36,37].

Multiple studies have shown that chewing abilities affect cognitive functioning [38,39]; however, very few studies have clinically measured chewing abilities and identified its correlations with ADLs, nutritional status, and cognitive disorder. This study focused on oral health, which can be managed and prevented over an individual’s lifetime, focusing especially on chewing ability. We have attempted to measure their direct effects on the cognitive functioning of elderly individuals and indirect effects that mediate decreases in ADLs and nutritional status, which are known to be preceding risk factors to dementia.

The average age of the participants was 81.4 years. Moreover, 162 participants were uneducated, and 105 had received only a primary school education. The average MMSE score was 23.1%, showing similar results as the average values for standard MMSE scores for those with a less than primary school education for elderly people aged 80–90 years suggested in the Report for Standardization of Dementia Measurements [18].

The MMSE scores relating to chewing ability were lower for groups with lower chewing abilities, revealing that the MMSE scores for those with a reduced chewing ability suggested diminished cognitive functioning relative to the other two groups’ functioning. The SEM analysis also indicated that chewing abilities influenced cognitive functioning, which was consistent with the results of Kimura et al. [40]. Moreover, these results are similar to those of existing studies that reported cognitive functioning disorders in elderly people with lower chewing abilities [41,42,43,44].

The ADL and IADL scores relating to chewing abilities were higher for groups with lower chewing abilities and were dependent and showed statistically significant differences. The SEM analysis also indicated that chewing abilities influenced ADLs, a finding that was similar to previous studies reporting the relationship between the number of chewable products and risks of dependency in daily living [27,40,41,42,43,44].

The MNA scores relating to chewing ability were lower for the groups with reduced chewing abilities, showing risks of nutritional disorders and statistically meaningful differences. The SEM analysis also indicated statistically significant effects, which matched the results of existing studies on the relationship between food chewing and oral health in elderly individuals [45,46,47].

Apart from chewing ability, ADLs and nutritional status, when checked for relationships with cognitive functioning, were found to be individually influential on cognitive functioning, which matched the results of existing studies [11,30,31,34,48,49].

Chewing ability also had a significant effect on cognitive functioning through nutritional status and ADL as mediating variables, showing that chewing ability directly and indirectly affects cognitive functioning.

One limitation of this analysis is the cross-sectional study, which limits the ability to analyze dynamics. Another limitation is the possibility of sampling bias and participation bias as the sample is not representative of the population of Korean elderly. Therefore, the results of this study should be interpreted with caution and should be complemented by the results of a larger study population. Further, a limitation of this study was that dental experts did not measure chewing ability. However, we have amended this through the evaluator training that we performed. We believe that individuals whom the elderly encounter for the first time would have received less honest responses compared with previously known home care nurses, leading to the possibility of more active evaluations.

## 5. Conclusions

This study measured the chewing abilities of the elderly; identified their cognitive functioning, ADLs, and nutritional status; and examined the relationships as well as direct and indirect influences. The study results are as follows:

1. When the participants were divided into groups with high, middle, and low chewing ability, the group with high chewing ability had significantly high ADL and IADL scores and were closer to dependency;
2. The MNA and MMSE-DS scores for the groups with low chewing ability were significantly lower;
3. The verification of the study model showed the presence of convergent, discriminant, and nomological validity; the model fit was in line with the standards of absolute and incremental indices of fit, which led to the conclusion that the study model and sample data were appropriate;
4. When we tested the hypotheses for direct effects, chewing abilities influenced cognitive functioning, ADLs, and nutritional status, and nutritional status directly affected ADLs;
5. When we tested the hypotheses for indirect effects, chewing ability indirectly affected cognitive functioning, with ADLs and nutritional status as mediators.

Despite the limitations of this study, the results demonstrate that chewing ability or oral health is certainly a very important factor in decreasing cognitive functioning. Oral health can be maintained through one’s lifetime; therefore, its effects can be strong compared with other disorders. Oral diseases are chronic and tend to accumulate. Maintaining oral health from much earlier on, before the effects of aging, can prevent decreased ADLs and cognitive functioning. Therefore, the importance of maintaining oral health is emphasized for elderly adults, and its influence will also affect families’ responsibility and the national economy. In the future, factors influencing the elderly’s chewing abilities such as saliva excretion; decreasing the number of remaining health, presence, and state of dentures; and prosthetic appliances should be researched to identify correlations and key factors that contribute to improving chewing abilities in an effective manner.

## Figures and Tables

**Table 1:** Participants’ general characteristics (*N* = 295).

		*n*	%
Sex			
	Male	95	32.2
	Female	200	67.8
Age (y) (range: 70–102 y, Mean ± SD: 81.35 ± 6.70)			
	70–74	54	18.3
	75–79	70	23.7
	80–84	73	24.7
	85–89	53	18.0
	≥90	45	15.3
Education			
	No formal education	162	54.9
	Primary school	105	35.6
	Middle school	16	5.4
	More than high school	12	4.1
Cohabitant			
	Yes	183	62.0
	No	112	38.0

**Table 2:** ADLs, MNA, and MMSE per chewing function.

	Range	Chewing Function				*p*-Value *
		Total	Low (Score 1–2)	Middle (Score 3)	High (Score 4–5)	
Chewing function *n* (%)						
	(1–5)	3.02 ± 0.02	100 (33.9)	86 (29.2)	109 (36.9)	
Activities of daily living						
ADL	(7–16)	7.45 ± 0.456	7.85 ± 0.856 ^a^	7.43 ± 0.436 ^a,b^	7.09 ± 0.096 ^b^	0.001
IADL	(10–33)	12.33 ± 2.33	13.73 ± 3.73 ^a^	12.69 ± 2.69 ^a^	10.77 ± 0.77 ^b^	<0.001
Nutritional assessment						
MNA	(9.5–30)	23.60 ± 3.60	22.73 ± 2.73 ^a^	23.01 ± 3.01 ^a^	24.86 ± 4.86 ^b^	<0.001
Cognitive functioning						
MMSE	(5–30)	23.14 ± 3.14	21.11 ± 1.11 ^a^	22.63 ± 2.63 ^a^	25.40 ± 5.40 ^b^	<0.001

Values are expressed as Mean ± SD. ADL, activity of daily living; IADL, instrumental activity of daily living; MNA, Mini-Nutritional Assessment; MMSE-DS, Mini-Mental Status Examination for Dementia. * Statistically significant by ANOVA (*p* < 0.05). ^a,b^ Significant difference between groups by Bonferroni correction.

**Table 3:** Model fit for the study model.

	*χ* ^2^	*χ*^2^/*df*	GFI	AGFI	CFI	RMR	RMSEA	TLI	NFI
Standard	*p* > 0.05	<3	>0.9	>0.9	>0.9	<0.05	0.1–0.08 normally 0.08–0.05 fine 0.05 > excellent	>0.9	>0.9
Study model	169.040 (*p* = 0.001)	2.037	0.933	0.903	0.954	0.102	0.049	0.942	0.915

**Table 4:** Path coefficient for study model.

	Path			Regression Weight	Standardized Regression Weight	SE	CR	*p*
H1	Chewing ability	→	Cognitive functioning	0.111	0.142	0.038	2.926	0.003
H2	Chewing ability	→	Activities of daily living	−0.218	−0.256	0.049	−4.490	<0.001
H3	Chewing ability	→	Nutritional assessment	0.156	0.173	0.053	2.925	0.003
H4	Activity daily living	→	Cognitive functioning	−0.645	−0.698	0.061	−10.590	<0.001
H5	Nutritional assessment	→	Cognitive functioning	0.103	0.118	0.044	2.359	0.018
H6	Nutritional assessment	→	Activities of daily living	−0.258	−0.272	0.056	−4.625	<0.001

H1: Chewing ability will affect cognitive functioning. H2: The ability to chew will affect the ability to perform everyday activities. H3: Chewing ability will affect nutritional status. H4: The ability to perform ADLs will affect cognitive functioning. H6: Nutritional status will affect the ability to perform daily activities. SE, standard error; CR, critical ratio.

**Table 5:** Direct, indirect, and total effects.

	Path			Direct Effect	*p*	Indirect Effect	*p*	Total Effect	*p*
H7	Chewing ability	→	Cognitive functioning	0.142	0.004	0.232	0.004	0.374	0.004
H8	Chewing ability	→	Activities of daily living	−0.256	0.004	−0.047	0.007	−0.303	0.004
	Chewing ability	→	Nutritional assessment	0.173	0.006	-	-	0.173	0.006
	Activity daily living	→	Cognitive functioning	−0.698	0.004	-	-	−0.698	0.004
H9	Nutritional assessment	→	Cognitive functioning	0.118	0.085	0.190	0.004	0.308	0.004
	Nutritional assessment	→	Activities of daily living	−0.272	0.004	-	-	−272	0.004

H7: Chewing ability will indirectly affect cognitive functioning through the activities of daily living, nutritional status parameters. H8: The mastication ability will indirectly influence the ability to perform daily activities through nutritional status parameters. H9: Nutritional status will indirectly affect cognitive functioning through the parameters of activities of daily living.

## Data Availability

Data are contained within the article.
